# Comparative effects of Yi Jin Jing versus Tai Chi exercise training on benign prostatic hyperplasia-related outcomes in older adults: study protocol for a randomized controlled trial

**DOI:** 10.1186/s13063-016-1448-4

**Published:** 2016-07-16

**Authors:** XiangYun Liu, Guoyuan Huang, Peijie Chen, Yong Li, JiuLin Xiang, Ting Chen, Ru Wang

**Affiliations:** Key Laboratory of Exercise and Health Sciences of Ministry of Education at the Shanghai University of Sport, 188 Hengren Road, Shanghai, 200438 China; Pott College of Science, Engineering & Education, University of Southern Indiana, Evansville, USA; FuDan University, 130 Dongan Road, Shanghai, 200032 China

**Keywords:** Benign prostatic hyperplasia, Mind–body exercises, Yi Jin Jing, Tai Chi, Comparative effectiveness research, Randomized controlled trial

## Abstract

**Background:**

Benign prostatic hyperplasia (BPH) and its associated lower urinary tract symptoms (LUTS) occur very commonly in older men. BPH and LUTS cause substantial physical and psychological impairment that could seriously affect the quality of late life and greatly cost the health-care systems. Current surgical and pharmacological therapies are expensive, may not effectively improve prostate function and health but cause adverse effects. There is an urgent need to find new and effective non-pharmacological preventions and treatments. Yi Jin Jing and Tai Chi are two common traditional Chinese mind–body exercises with different movements and techniques, but both emphasize regulating functional homeostasis and keeping whole body harmony. Yi Jin Jing and Tai Chi have not been studied much for potentially use in the treatment of BPH-related problems. The primary purpose of this protocol is to assess the effectiveness of Yi Jin Jing versus Tai Chi on the monographic and functional changes of prostate in older men.

**Methods/Design:**

A prospective single-center randomized controlled trial will be conducted. A total of 150 old men (60–70 years old) will be recruited from the urban tertiary of Shanghai, China. Of these, 50 eligible participants will be randomly assigned to a control group and two intervention groups with either Yi Jin Jing or Tai Chi exercise training. They will undergo 30 minutes for each exercise for five times a week for 6 months. The primary outcomes are changes of signs and symptoms in BPH and lower urinary tract from baseline to post-intervention. The main secondary outcomes are exercise-induced effects on the circulating levels of estrogen and androgen. All the outcome measures will be assessed at baseline, immediately after the 6-month intervention, and at the 3-month post-intervention follow-up.

**Discussion:**

This proposed study will be the first comparative randomized clinical trial to evaluate the effectiveness of Yi Jin Jing versus Tai Chi exercise on prostate health among older adults. The results will provide an evidence-based recommendation for Chinese older men on the use of Yi Jin Jing and Tai Chi training to promote prostatic function and health. Potential mechanisms for the regulatory effect of the two exercises elucidated by multiple outcomes are also explored. A clarification of the effects and mechanisms may provide information for the development of new strategies in the prevention and treatment of BPH-related conditions.

**Trial registration:**

ClinicalTrials.gov Identifier: ChiCTR-IOR-16007698. This trial was registered on 4 January 2016.

## Background

Known as a benign and non-cancerous enlargement of the prostate gland, benign prostatic hyperplasia (BPH) with its associated lower urinary tract symptoms (LUTS) is extremely common among male individuals. The number of BPH patients increases each year and it has become one of the major health concerns. The prevalence of BPH is highly correlated with age and thus, BPH-related conditions occur more commonly among older males [1–3]. Recent statistics from China, for example, have shown that an estimated 62.9 % of men over the age of 50 have been diagnosed with BPH in Pudong New District of Shanghai. The prevalence of BPH may accelerate with aging, and the incidence can increase to 73.9 % and 84.2 % of older individuals in their seventh and eighth decades, respectively [4]. BPH interferes with the normal flow of urine and leads to many LUTS, including urinary hesitancy, frequent urination, urgency, thin urine flow, and urinary retention [5–7]. As a result, BPH can greatly affect the patients’ physical and mental health and have devastating effects on quality of life, plus there is loss of productivity and increased health-care costs.

Fast emerging as a major public health concern, scientific research on the prevention and treatment of BPH has become critical and has attracted significant attention, particularly for older men. However, the causes of BPH are complex and the overgrowth of the prostate gland is multifaceted involving various factors such as aging, hormones, late activation of cell growth, genetic, and lifestyle elements [8, 9]. Benefits have been found for current BPH treatment options including medication, minimally invasive therapies, and prostate surgery with continued surveillance [3, 10]. Unfortunately, current surgical and pharmacological therapies have limitations. For example, the drugs and surgeries are expensive, may not effectively improve prostate function and health, and may carry a hidden cost of significant risk of serious adverse events. Thus, there is an urgent need to find new and effective non-pharmacological preventions and treatments for individuals, especially older adults, with and without BPH and associated LUTS.

Importantly, accumulating evidence indicates that the development and progression of BPH and prostate cancer are closely related to a sedentary lifestyle. For example, the incidence of prostate cancer can be reduced by physical activity (PA) and exercise training [11–14]. More physically active men have a lower frequency of BPH and associated LUTS [15–17]. Occupational PA is inversely associated with BPH [18]. Exercise can effectively reduce the risks of symptomatic BPH and LUTS [19].

As a common and important mind–body (MB) exercise, Tai Chi interventions have been reported as having effective influence on health problems and chronic diseases, including fibromyalgia [20], type-2 diabetes mellitus [21], stroke [22], knee osteoarthritis [23], Parkinson’s disease [24–26], chronic obstructive pulmonary disease [27, 28], and BPH [29, 30]. There has not, however, been much research on Tai Chi training and BPH with associated LUTS. Only one randomized controlled trial, to our knowledge, has tried studying the topic [29]. This short-term study suggests that 12-week Tai Chi training may improve LUTS and quality of life in elderly patients with BPH, but the limitations and difficulties encountered in this study elicit a significant need for more prospective clinical trials.

Like Tai Chi, Yi Jin Jing is an attractive traditional Chinese MB exercise that is quickly emerging in China and is getting more and more attention due to its effects on health and physical fitness [31–33]. In recent years, Yi Jin Jing exercise training has been widely applied to improve physical functions, to promote health, and to prevent or treat disease-related conditions and physical disabilities. Some reported benefits of Yi Jin Jing practice include enhanced dynamic nervous response speed [34]; decreased Quetelet index and improved lung capacity [35]; improved body serum superoxide dismutase activity and decreased serum malondialdehyde (MDA) levels [36, 37]; delaying the decline of intelligence by improving thinking agility, action time, and short-term memory [38]; and improvements of psychological adjustment capability and depression [39–41].

To our knowledge, no research has been conducted on Yi Jin Jing training, BPH and its associated LUTS. The preliminary results of our pilot study on rat models presented a three-dimensional relationship between androgen levels and prostate volume [42]. However, the relationship for the role and effect of Yi Jin Jing training in mediating hormone regulation is not known. It is still unclear if this type of exercise provides health benefits that will prevent BPH and is therapeutic for patients with BPH and LUTS. Accordingly, we designed this randomly controlled trial with the specific purpose of investigating the effectiveness of a 6-month Yi Jin Jing exercise program on prostate function and health in older individuals, determining the direction and magnitude of Yi Jin Jing exercise-induced changes (including secretion of the sex hormone androgen and prostate volume), and exploring the molecular mechanisms that mediate these effects of Yi Jin Jing exercise on BPH.

## Methods/Design

### Study design

**Fig. 1:**
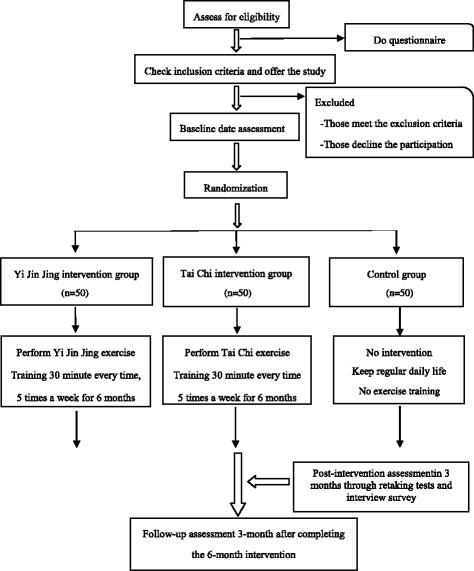
Study design as flow diagram: recruitment, randomization, intervention, follow-up, assessment, and outcomes

To examine the effectiveness of the Yi Jin Jing exercise intervention on older adults, compared with Tai Chi training, we designed this study as a single-center repeated-measures randomized controlled clinical trial. Old subjects will be recruited from the community of Shanghai, China. With a 1:1:1 allocation ratio, enrolled eligible volunteers will be randomly assigned to three parallel arms including two intervention groups and a non-intervention control group (*n* = 50 per group). Participants in the two intervention groups will train with Yi Jin Jing or Tai Chi exercise continually for 6 months, while the control group will not receive any intervention. Assessments of the primary and secondary outcomes and data analysis will be performed at baseline, immediately after the 6-month intervention, and at the 3-month post-intervention follow-up. All tests and measurements will be conducted by trained professionals who are blinded to the group assignment of subjects and their specific background. The flow of participants through the trial is shown in Fig. 1.

### Sample size

The sample size was estimated using a randomized controlled trial about Tai Chi for LUTS and BPH [29] and we expanded the sample size to make it adequate to detect the expected changes of prostate volume. Ultrasound will be used to measure these changes before and immediately after the 6 months of Yi Jin Jing exercise training, and at the 3-month post-intervention follow-up. It is estimated that a sample size of 40 participants per group will be required to observe a similar result with a power of 80 %. Considering a 20 % dropout and exit rate, we will recruit 150 subjects with 50 in each group.

### Participant recruitment and selection

Participants are recruited by word of mouth, flyers, email lists, organization or club meetings, and other relevant activities from the general population in Shanghai, China. The first step is to use an interview to recruit the potential older individuals who are interested in participation in the research. Potential participants 60–70 years old are invited and screened using a RAND-36 questionnaire [43, 44]. After completing the questionnaire screening, the interested and eligible participants are asked to attend an information meeting. They are provided with a written informed consent form to be signed and they continue with the further steps, beginning with a more detailed health screening and physical assessment.

### Inclusion criteria

Participants are required to meet all the following criteria:

1. Aged between 60 and 70 years
2. A relatively stable body weight over the past 6 months with a weight change of less than 2 %
3. A sedentary lifestyle, not having exercised for the past 3 months, or exercising occasionally less than once a week for 20 min each time
4. No Yi Jin Jing and/or Tai Chi training experience for the past 3 months
5. Apparently healthy, free of cardiovascular disease, and not being treated with BPH drugs or other therapies during the last 6 months
6. Voluntary participation in this research for at least 6 months and provision of written informed consent

### Exclusion criteria

Subjects are excluded for any of the following:

1. Signs and symptoms of or having the following diseases (but not limited to these diseases): Crohn’s disease, sarcoidosis, chronic diarrhea, heart disease, rheumatoid arthritis, systemic lupus erythematosus, and cancer
2. Having taken any relevant drugs in the last 6 months, such as for insulin-dependent diabetes mellitus or antibiotics
3. Any conflict with the objectives of this study

### Randomization

After providing written informed consent, eligible participate will be randomly assigned into a control or one of two intervention groups, which have equal numbers of subjects. The randomization procedure will be conducted by an independent statistician using a computerized randomization program. Participants and three principal researchers are aware of the study-group assignments. However, to minimize any potential bias, other study personnel, especially those performing the tests and outcome evaluators or adjudicators, will be blinded to the subjects’ intervention allocation.

### Intervention

The subjects in both intervention groups will undergo the Yi Jin Jing or Tai Chi exercise training, respectively, with 30-minute sessions five times a week for 6 months. The control group will keep their regular daily life in normal living conditions without any intervention and/or exercise training. After completing the 6-month treatment sessions, participants in the Yi Jin Jing and Tai Chi groups will be asked to continue with their exercises, while the control participants are encouraged to maintain their usual activities and life. The research team will monitor these participants twice a month with home calls until the 3-month follow-up evaluations. All exercise participants are asked to record their exercise behavior for the 3 months.

All measurements will be conducted for every participant at baseline 2 weeks prior to starting the intervention, after completing the intervention (by retaking the interview surveys and tests in the sixth month), and at the 3-month post-intervention follow-up. Fasting blood samples will be collected in the morning of the testing day. In particular, we will measure prostate volume by ultrasound at baseline, at the end of the 6-month intervention, and additionally at the 3-month post-intervention follow-up. The intervention will be closely supervised by a physician and study personnel to minimize the occurrences of any accident or injury.

### Attrition and compliance

Due to the voluntary nature of the enrollment and that the subjects are older adults, the attrition rates of this study may be low. Considering other factors such as environment, health reasons, or other emergency situations, we expect a general attrition rate of 20 % in this study. Our recruitment plan takes this into account. To maximize compliance, retention in the study and adherence to treatment will be monitored prospectively and routinely throughout the trial.

### Study measurements

A structured interview and surveys will be used for the three groups at pre-intervention, post- intervention, and the follow-up to obtain self-reported outcomes. Although the participants will be aware of their study-group assignments, we will use the same format for interviews and surveys in the three groups to minimize ascertainment bias. Medical records will be obtained for their documentation of events. Whenever researchers become aware of an injury or accident, a standard protocol will be used to obtain information on the event. Demographic data will be collected at baseline. Clinical and laboratory measurements are performed at 2 weeks before starting the 6-month intervention (baseline), 1 week after completing the 6-month intervention, and at the 3-month follow-up. We also plan to compare the effects and outcome changes induced by Yi Jin Jing versus Tai Chi training on BPH.

### Study outcomes

Demographic information of the subjects will be collected, including age, body weight, body composition, resting heart rate, and resting blood pressure. The physical fitness of each subject will be tested and evaluated prior to and post the intervention. The primary outcome will be the exercise-induced changes of:

1. Prostate volume, which will be measured using a Madison SA600P portable B ultrasound device
2. BPH and LUTS symptoms, which will be measured using the international prostate symptom score (IPSS)
3. Urination-related quality of life (Qol) before and after the intervention and at the follow-up

IPSS is identical to the American Urology Association (AUA) symptom index, which was developed by AUA in 1991. IPSS and Qol have been translated into many languages and are widely used around the world. The secondary outcomes are the blood chemical analyses of exercise-induced changes, which will be obtained at baseline, post-intervention, and at the 3-month follow-up. They are the circulating levels of fasting blood samples (2 mL, 12-hour fasting) and serum levels of sex hormones including dihydrotestosterone, progesterone, estrogen, testosterone propionate, and androgen, which will be measured using chemiluminescent magnetic enzyme-linked immunoassay systems. All the assessments will be performed in the laboratories of the Exercise Science College at the Shanghai University of Sport.

### Data management and statistical analysis

The baseline and later data will be collected and recorded after careful checking. Both quantitative and qualitative data, including the volume of the prostate, IPSS, Qol, and the level of sex hormones (dihydrotestosterone, progesterone, estrogen, testosterone propionate, and androgen), will be numerically coded and analyzed on an intention-to-treat basis. Participants who withdraw will be treated as having no change from baseline at all times after dropping out. Group differences at baseline will be tested using a two-sample *t*-test for quantitative data and a chi-square test for qualitative data. Repeated-measures analysis of variance (ANOVA) and Mann-Whitney *U* test analysis for each of the outcome variables will be conducted to determine the treatment effects in terms of within-group, between groups, and interactive group-by-time modes. Prior to further statistical analysis, parametric assumptions of normality and homoscedasticity for each variable will be checked using standard tests and graphical methods. According to the serum sex hormone test results, we will use MATLAB software to analyze the changes of serum E2/T ratio. This will probably allow us to develop further the fitted equation of the linear regression and create the three-dimensional graphics between the androgen levels and BPH for the changes in the measured scores before and during the Yi Jin Jing and Tai Chi exercise training, immediately on completing the intervention, and the follow-up. All statistical analyses will be performed using the Statistical Package for the Social Sciences (SPSS, Inc., Chicago, Illinois, United States) for Windows version 18.0. Unless otherwise noted, all data will be reported as mean ± SD. A level of *p ≤ 0.05* will be used for statistical significance.

## Discussion

Current treatments of BPH to improve LUTS are mainly based on surgery and anti-hormone drugs [45, 46]. Unfortunately, these therapies cause a considerable number of side effects, such as the risk factors associated with prostatic surgery [47, 48] and the drug-related problems for patients with BPH due to the anti-hormone drugs [49]. These data imply that there are considerable limitations in the surgical treatments and pharmacotherapy. Thus, exploring more effective therapeutic methods as independent and/or supplementary therapeutic modalities is clinically meaningful for the treatment and management BPH and LUTS.

Lifestyle modifications are beneficial for reducing the risk of developing BPH and LUTS [8, 50, 51]. PA may provide cost-effective health benefits other than those of pharmacotherapy or surgery for the treatment or rehabilitation of BPH [10–13]. MB exercise as an important approach in complementary and integrated medicine may facilitate the mind’s effect on bodily functions and promote health and well-being. Tai Chi is considered to be effective in improving integrative health and has the advantages of being simple, convenient, efficient, and inexpensive, without severe adverse effects. There is very limited research on Tai Chi training and BPH. Although a previous study reported that 12 weeks of Tai Chi training may have an effect in elderly patients with BPH, the intervention failed to prove the hypotheses in terms of the possible mechanisms including improved LUTS of BPH, a decreased resting sympathetic tone in the prostate, and an alteration in the levels of certain hormones [29]. Accordingly, further prospective investigations are needed to elucidate Tai Chi’s effect on BPH and the associated LUTS.

Emerging very quickly in China in recent years, Yi Jin Jing is becoming an attracting MB exercise like Tai Chi. A limited number of studies, though the quality of these studies is questionable, have reported the positive effect of Yi Jin Jing training on improving functional capacity and potentially reducing the incidence of diseases [35–38]. There has been very limited research in this area, and these crude results were unfortunately observed in non-randomized controlled trials or the research presented is lacking in quality. These studies have significant limitations, such as methodological issues and small sample sizes. Thus, additional prospective randomized controlled clinical trials with large samples and well-designed strict systematic operation, and high-quality practice are needed to evaluate the effectiveness of Yi Jin Jing exercise on BPH and LUTS in older individuals.

To the best of our knowledge, this is the first study to evaluate the effectiveness of Yi Jin Jing versus Tai Chi training on prostate function and health in a large older population. This trial will determine the effects of a 6-month Yi Jin Jing training program on adaptive changes of the prostate, including anthropometric, physiological, and biochemical parameters, in older men. We will also compare Yi Jin Jing and Tai Chi, as two important MB exercises, regarding their preventive and/or therapeutic benefits on prostate health. This trial will evaluate monographic and functional changes induced in the prostate by Yi Jin Jing and Tai Chi through monitoring a wide spectrum of health indices, including blood pressure, blood serum biochemical test, hematology indices of sex hormones (estradiol, testosterone, double hydrogen, and androgen), and an ultrasound of the prostate. Thus, the measured and analyzed data will allow us to explore possible cellular and molecular mechanisms that mediate these effects of Yi Jin Jing compared to Tai Chi on BPH, such as by examining the corresponding changes in sex hormones levels and through an advanced analysis of the serum E_2_/T ratio, a mathematical simulation of serum E_2_/T ratio, and the prostate viscera coefficient. Importantly, no studies have considered if long-term and short-term interventions of Yi Jin Jing versus Tai Chi exercise have a different role in mediating the hormone regulatory effect on BPH. Accordingly, we are expecting to learn whether participants receiving different periods of supervised Yi Jin Jing and Tai Chi training (12 weeks, 24 weeks, and 36 weeks) have different improvements in the biomarkers measured.

Successful completion of the proposed study will contribute to the evidence-based knowledge of whether Yi Jin Jing and Tai Chi are clinically favorable and preferable as simple, inexpensive, effective, and durable prevention and/or treatment approaches for the major disabling disease BPH, which incidentally may decrease the economic costs to the health-care system. The results of this study may provide valuable information for the development of new strategies in preventing and/or treating BPH and the associated LUTS and thus, they may have important public health implications for older adults with/without such chronic conditions.

## Trial status

A pilot study involving a small group of mild BPH in old men has been completed at our institution. Recruitment will only begin at future sites once all necessary local approvals have been granted. Baseline measurements will be taken in February 2016, and the 6-month intervention will begin in March 2016. The trial is due to be completed in December 2016.

## Abbreviations

AUA, American Urology Association; BPH, benign prostatic hyperplasia; IPSS, international prostate symptom score; LUTS, lower urinary tract symptoms; MB, mind–body; MDA, malondialdehyde; PA, physical activity; Qol, quality of life
